# Idiopathic Central Precocious Puberty and Attention Deficit Hyperactivity Disorder: A Clinical Case Study

**DOI:** 10.1192/j.eurpsy.2025.1151

**Published:** 2025-08-26

**Authors:** G. Juárez

**Affiliations:** 1 Psiquiatria, Hospital Universitario La Paz, Madrid, Spain

## Abstract

**Introduction:**

Precocious puberty is defined as the appearance of secondary sexual characteristics before the age of 8 in girls and 9 in boys. It can be classified into central precocious puberty (dependent on the hypothalamic-pituitary-gonadal axis) and peripheral precocious puberty. Attention Deficit Hyperactivity Disorder (ADHD) occasionally co-occurs with precocious puberty, complicating its management. This case focuses on a 9-year-old girl diagnosed with idiopathic central precocious puberty, treated with both GnRH analogues and stimulant medication, showing significant improvement in symptoms.

**Objectives:**

The primary objective is to explore the relationship between these two conditions through the presented clinical case. Additionally, the study aims to evaluate the impact of hormone-suppressing medications and ADHD treatment on the patient’s social and academic functioning.

**Methods:**

A 9-year-old girl with precocious puberty and ADHD symptoms, diagnosed through hormonal tests and ADHD scales administered to parents and teachers, as well as clinical assessments of emotional status. A multidisciplinary approach is essential for managing complex cases involving idiopathic central precocious puberty and ADHD. Follow-up was conducted every 3 months to assess pubertal status, ADHD symptoms, and psychosocial adjustment.

**Results:**

GnRH analogue therapy successfully arrested pubertal progression, with bone age stabilization and normalized growth velocity.ADHD symptoms improved significantly with extended-released methylphenidate, leading to better attention, reduced hyperactivity, and improved classroom behavior.The patient demonstrated enhanced self-esteem, better peer relationships, and a positive psychosocial outlook.

**Conclusions:**

The relationship between ADHD and precocious puberty may be due to a combination of hormonal, neurobiological, and psychosocial factors. Although the precise connection has not been fully determined, there appears to be an interaction between the neuroendocrine system and brain pathways that regulate behavior and development.

**Disclosure of Interest:**

None Declared

